# Assessing Anticoagulation in Neonates With Congenital Diaphragmatic Hernia During Extracorporeal Membrane Oxygenation: Does Anti-Factor Xa or Thromboelastometry Provide Additional Benefit?

**DOI:** 10.3389/fped.2021.685906

**Published:** 2021-09-17

**Authors:** Alba Perez Ortiz, Carl E. Dempfle, Toni Jung, Thalia Doniga, Christel Weiß, Svetlana Hetjens, Thomas Schaible, Neysan Rafat

**Affiliations:** ^1^Department of Neonatology, University Children's Hospital Mannheim, University of Heidelberg, Mannheim, Germany; ^2^IMD Coagulation Center Mannheim, Mannheim, Germany; ^3^Department for Medical Statistics and Biomathematics, Medical Faculty Mannheim, University of Heidelberg, Mannheim, Germany

**Keywords:** extracorporeal membrane oxygenation, congenital diaphragmatic hernia, thromboelastometry, anti-factor Xa, heparin

## Abstract

**Objective:** The optimal management of anticoagulation in neonatal/pediatric patients during extracorporeal membrane oxygenation (ECMO) has not been established yet and varies greatly among ECMO centers worldwide. Therefore, we aimed to assess whether the use of anti-factor Xa assay and/or thromboelastometry correlate better than activated clotting time with heparin dose in newborns with congenital diaphragmatic hernia during ECMO. We also examined whether these coagulation assays correlate with thrombotic and/or hemorrhagic complications, when the management of anticoagulation is based only on activated clotting time values.

**Methods:** A prospective observational study in a neonatal ECMO center was conducted. We included all neonates with congenital diaphragmatic hernia born in our institution between March 2018 and January 2019 and requiring support with venoarterial ECMO. A total of 26 ECMO runs were analyzed. During the study, the heparin dose was still adjusted according to activated clotting time values. Measurements of anti-factor Xa assay, activated partial thromboplastin time, and a thromboelastometry from the same blood specimen were performed twice a day.

**Results:** Anti-factor Xa levels showed a moderate correlation with heparin dose, whereas the other tests showed a weak correlation. Four patients (17.4%) had thrombotic complications, 2 patients (8.7%) experienced life-threatening bleeding, and in 11 patients (47.8%) disseminated intravascular coagulation (DIC) occurred. Anti-factor Xa levels were lower in the group with thrombotic complications (0.23 vs. 0.27 IU/ml; *p* = 0.002), while activated partial thromboplastin time was higher in the group with hemorrhagic complications (69.4 s vs. 59.8 s; *p* = 0.01). In patients experiencing DIC, heparin dose and anti-factor Xa levels were lower, while no difference in activated clotting time and clotting time in INTEM and INTEM-HEPTEM were shown.

**Conclusions:** Anti-factor Xa levels correlate better to heparin dose than activated clotting time. The use of anti-factor Xa assay instead of activated clotting time for dosing of unfractionated heparin could reduce thrombotic complications in neonates with congenital diaphragmatic hernia on ECMO support. The thromboelastometry showed no additional benefit for this purpose.

## Introduction

Extracorporeal membrane oxygenation (ECMO) is an extracorporeal technique used to support patients with respiratory and/or cardiac failure refractory to conventional treatment. Since the first reported use of ECMO in a newborn in 1975, over 40,000 newborns worldwide have been treated with ECMO (1). Congenital diaphragmatic hernia (CDH) is nowadays the most common indication for ECMO in neonates with respiratory failure (2).

Despite increasing clinical practice and technical improvements, the optimal management of anticoagulation in patients during ECMO is still controversial and varies greatly among the various ECMO centers worldwide (3). The rate of hemostatic complications, including life-threatening bleeding and thrombotic events, remains high (10–33%) and represents a major cause of morbidity and mortality in these patient populations (1, 3, 4). Maintaining an optimal balance of hemostasis can be particularly challenging in neonates due to their immature hemostatic systems, with decreased plasma concentrations of most procoagulants and many anticoagulant factors and the resulting lack of reserve capacity (1, 4). Furthermore, these physiological differences may influence the monitoring and efficacy of the anticoagulation required during ECMO (1).

Although there is no standardized protocol, unfractionated heparin (UNFH) is the most commonly used anticoagulant during ECMO (1, 3, 5). Several tests to monitor coagulation and anticoagulation during ECMO have been developed over time, but the ideal test or combination of tests to adequately manage anticoagulation still remains to be established (1, 6–9).

The activated clotting time (ACT) is the most frequently used test to monitor UNFH activity in patients supported on ECMO, because it is available in real time at the bedside (4, 6). However, recent studies suggest that managing ECMO anticoagulation solely based on ACT measurements may lead to sub-optimal anticoagulation in these patients (4, 9–12). The activated partial thromboplastin time (aPTT) is the second most frequently used test to monitor UNFH during ECMO (1). The anti-factor Xa assay does not provide a direct measurement of the UNFH concentration but of the UNFH effect instead (6). Its value appears to be more specific than ACT values for heparin control, as it is not affected by coagulopathy, thrombocytopenia, coagulation factor deficiency, or dilution (6). Thromboelastometry (ROTEM^®^ Tem Innovations GmbH, Munich) is a whole blood viscoelastic point-of-care coagulation test that provides information on the overall kinetics of hemostasis, as well as on the interactions between coagulation factors, erythrocytes, platelets, and coagulation inhibitors (6). Such information makes the use of viscoelastic tests in ECMO patients compelling.

The objectives of this study were to examine the correlations between ACT, aPTT, anti-factor Xa assay, and two ROTEM^®^ parameters that detect the UNFH effect (CT-INTEM and CT-INTEM–CT-HEPTEM) and the dose of UNFH in neonates with CDH during venoarterial ECMO. Furthermore, we were interested in assessing whether the different measurements of anticoagulation correlate with thrombotic and/or hemorrhagic complications when the management of anticoagulation is based only on ACT values.

## Materials and Methods

### Subjects

Neonates with CDH requiring venoarterial ECMO (*n* = 23) were selected directly after birth between March 2018 and January 2019 from the neonatal intensive care unit (NICU) of the Department of Neonatology of the University Children's Hospital Mannheim, University of Heidelberg. The indication for and the allocation to ECMO was performed based on the recommendations made by the CDH EURO Consortium Consensus Team (2015 Update) (13). All gestational ages were included. Exclusion criteria were congenital heart defects (except patent ductus arteriosus and persistence of the foramen ovale), inborn errors of metabolism, other anatomical pulmonary anomalies, and severe pneumonia/sepsis. We selected only CDH patients in order to have a homogenous patient population for this study. This study was approved by the local ethics committee of the Medical Faculty Mannheim of the University of Heidelberg (Ethics Committee II), and informed consent was obtained from the parents of all study subjects.

### Extracorporeal Membrane Oxygenation and Anticoagulation

All patients received an ECMO circuit consisting of a Jostra HL20 roller pump (Maquet Cardiopulmonary AG, Hirrlingen, Germany), a QUADROX-i Neonatal Oxygenator (Maquet Cardiopulmonary AG, Hirrlingen, Germany), and a heparin-coated system Maquet Bioline (Maquet Cardiopulmonary AG, Hirrlingen, Germany). Anticoagulation was performed with a continuous infusion of UNFH. The circuits were all primed with gas (carbon dioxide) following a prime with Ringer's solution with UNFH (0.5 IU/ml), an albumin prime (50 ml 20% Albumin), and finally a blood prime with one packed red blood cells, 50 ml fresh-frozen plasma, and 200 IU UNFH. During ECMO circuit prime, a continuous infusion of UNFH at 500 IU/h was started. After finishing the blood prime, an ACT measurement was taken, and if it was greater than 999 s, the UNFH infusion was reduced to 300 IU/h until connection to the patient. No bolus of UNFH was given to patients prior to cannulation. After cannulation and start of ECMO, continuous infusion of UNFH was decreased to 100 IU/h and gradually reduced until ACT values were within target range (160–180 s). During the study, UNFH was still titrated according to ACT values measured hourly. Target range from ACT was increased from 160–180 to 180–200 s in case of the presence of thrombosis in ECMO circuit. According to our protocol, antithrombin (AT) activity was measured daily, and values below 60% were systematically supplemented. Transfusion requirement was monitored twice a day: platelet transfusions (15 ml/kg) were administered to achieve platelet counts higher than 80,000 cells/μl during the first 72 h of ECMO and greater than 60,000 cells/μl thereafter. Fresh-frozen plasma (15 ml/kg) was also administered to keep fibrinogen level above 100 mg/dl and Quick test value higher than 40%.

### Monitoring of Anticoagulation

Measurements of ACT, anti-factor Xa assay, aPTT, and thromboelastometry from the same blood specimen were performed twice a day (7 a.m. and 7 p.m.). The ACT was measured at the bedside using ACT Plus^®^ (Medtronic Biologic Therapeutics and Diagnostics, Minneapolis, USA). Both aPTT and anti-factor Xa were collected in citrated tubes and measured at the Institute of Clinical Chemistry of the University Hospital Mannheim. The aPTT was measured using a coagulometric test with addition of Actin FS and calcium chloride (Sysmex^®^ CS-5100, Siemens Healthcare Diagnostics Products GmbH, Marburg, Germany). The assessment of anti-factor Xa was performed with a chromogenic test without addition of exogenous ATIII (INNOVANCE^®^ Heparin test, Siemens Healthcare Diagnostics Products GmbH, Marburg, Germany). The thromboelastometry was performed as a point-of-care examination using ROTEM^®^ delta (Tem Innovations GmbH, Munich, Germany). In each ROTEM^®^ analysis, four tests were performed simultaneously: EXTEM, INTEM, FIBTEM, and HEPTEM. Since the effect of heparin can be specifically demonstrated by comparing INTEM (reflects the intrinsic coagulation pathway) and HEPTEM (it includes heparinase and reflects the intrinsic coagulation pathway in the absence of UNFH), both the coagulation time for INTEM (CT-INTEM) and the difference between the coagulation time for INTEM and HEPTEM (CT-Diff) were used to analyze the heparin effect.

### Data Collection

Demographic, clinical, and laboratory data were collected, including demographic variables, duration of ECMO support, UNFH dosing, transfusion requirement, the presence of disseminated intravascular coagulation (DIC), and thrombotic and hemorrhagic complications as defined by the Extracorporeal Life Support Organization (ELSO) registry (6). In the absence of ECMO-specific, validated DIC scores (14), DIC was defined as a composite of thrombocytopenia (<50,000 cells/μl), d-dimer >20 g/l, fibrinogen <100 mg/dl, and Quick test <39%. Laboratory values were collected twice a day and included platelet count, hemoglobin, ACT, Quick test, aPTT, fibrinogen, anti-factor Xa, and ROTEM^®^ values. AT and d-dimer levels were measured once a day.

As described above and according to our protocol, the solution and the blood products used for the priming of the ECMO circuit contained UNFH, and a continuous infusion of UNFH (100 IU/h) was started after cannulation. Due to this high infusion rate of heparin, the values of the coagulation assays were outside the range of measurement in the first hours after initiation of ECMO support, and the UNFH infusion rate had to be significantly reduced until ACT values were within the target range. To guarantee that this situation did not affect the results, these outliers of the values from the coagulation tests (ACT levels >200 s in the first 2–12 h after cannulation until ACT target range of 180–200 s was reached for the first time) as well as the corresponding UNFH dose and associated bleeding complications (such as cannula site bleeding directly after cannulation) were excluded from the statistical analyses.

### Statistical Methods

Statistical analysis was performed with SAS^®^ Version 9.4 (SAS Institute GmbH, Heidelberg, Germany). Descriptive statistics were used to describe the demographic characteristics of the patients and to analyze the distribution of UNFH infusion rates and coagulation tests. Each ECMO course was divided into 12-h periods daily, and each laboratory value and UNFH infusion rate was recorded within its corresponding time point. Spearman correlation coefficients were used to assess correlations between heparin dose and values from the different coagulation assays. The Wilcoxon–Mann–Whitney test was used to compare the values from the coagulation assays and the heparin dose between groups with and without clinical complications. A *p*-value of 0.05 or less was considered significant.

## Results

### Demographic and Clinical Characteristics of the Study Cohort

Twenty-three newborns (7 female and 16 male) were included in our study, of which two patients (8.7%) were late preterm infants (34^+5^ and 36^+0^ weeks). The remaining patients were term neonates (37^+1^-39^+4^ weeks). The median birth weight was 2,894 g (range 2,160–3,890 g). All patients were placed on ECMO in the first 48 h after birth. After 10 days, three patients required a second ECMO run; hence, a total of 26 ECMO runs were analyzed. The median duration of ECMO support was 10.3 days (range 1–20 days). Nine patients (39.1%) died prior to discharge, four of them (17.4%) died while on ECMO support.

With regard to hemostatic complications, four patients (17.4%) showed significant thrombosis in the ECMO circuit, requiring an unplanned replacement of the circuit or an unplanned termination of the ECMO support. No patient experienced a clinically noticeable thrombotic complication. Eight patients (34.8%) had hemorrhagic complications, of which six developed immediate bleeding at cannulation site, and three required surgical revision. Two patients (8.7%) experienced life-threatening hemorrhagic complications (one pulmonary and one cerebral) after the first 24 h of ECMO support. None of the patients experienced lethal bleeding. DIC occurred in 11 patients (47.8%).

### Monitoring of Anticoagulation

**Figure 1:**
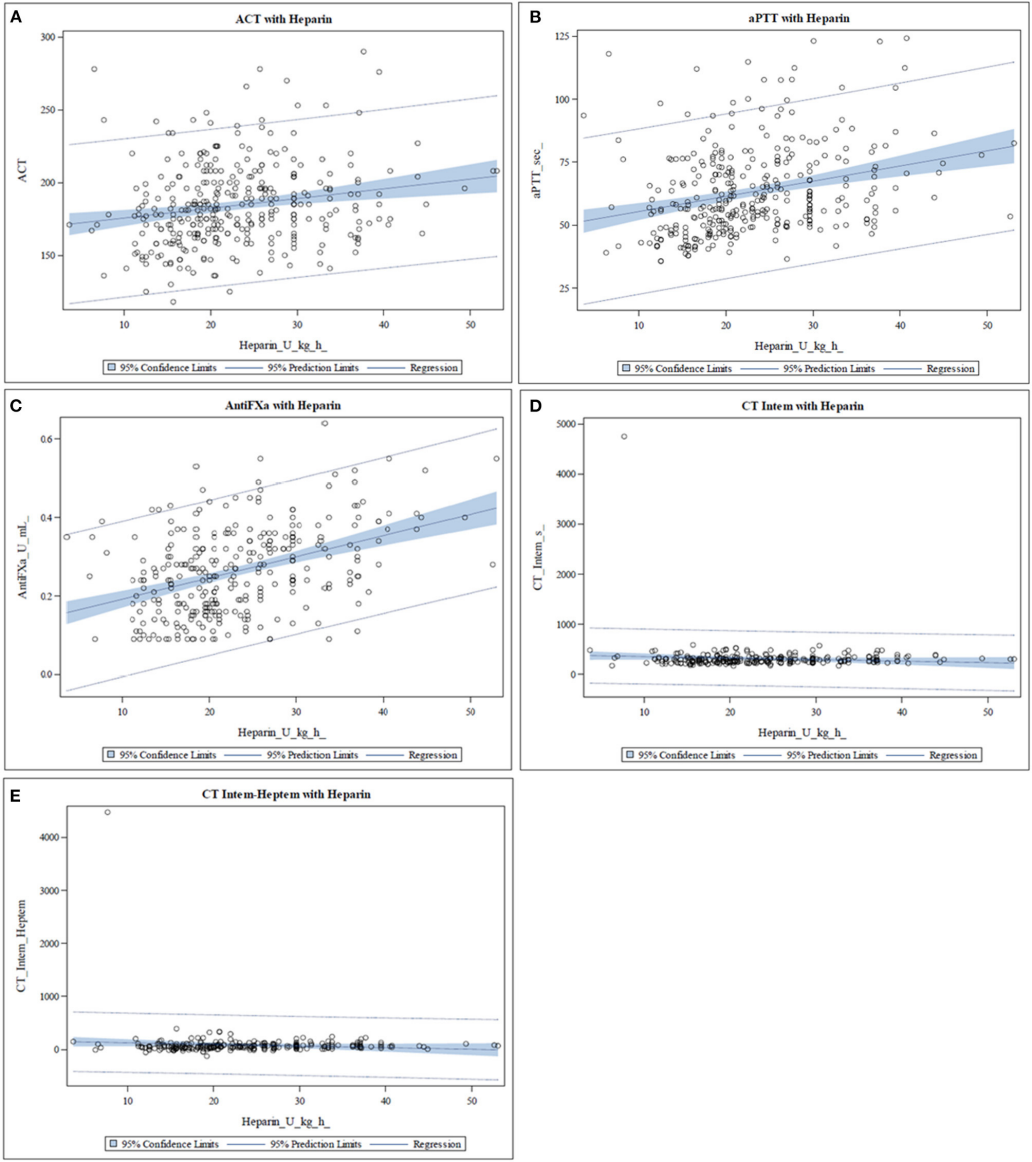
Scatter plots representing the relationship between heparin dose and the different coagulation assays. The blue area represents the 95% confidence limit of the mean predicted values of the regression line. (**A–C**) The values of activated clotting time (ACT), activated partial thromboplastin time (aPTT), and anti-Xa assay increase with increasing heparin dose. In the case of anti-Xa assay, fewer outlying values and a steeper regression line are seen. (**D,E**) The slightly slanted horizontal line indicates a lack of sensitivity of the clotting time in INTEM (CT-INTEM) and INTEM-HEPTEM (CT-Diff) in response to a change in heparin dose.

The median values of the different coagulation tests are shown in Table 1. None of the coagulation assays showed a strong correlation with the heparin dose (Table 2). Anti-factor Xa assay showed a positive moderate correlation with the heparin dose, whereas the other tests correlated only weakly (Table 2). No significant correlation was found between heparin dose and CT-INTEM (Table 2). In Figure 1, the correlation of heparin dose to the values from the analyzed coagulation tests are displayed. The gently slanted horizontal lines shown in Figures 1D,E indicate a lack of sensitivity of the analyzed thromboelastometric tests in response to a change in heparin dose. In contrast, an increase in heparin dose increased the values of ACT, aPTT, and anti-Xa assay (Figures 1A–C), while the steeper regression line and the fewer outlying values in the case of anti-Xa assay indicated a stronger correlation of this test to heparin dose in comparison with ACT and aPTT.

**Table 1:** Median values of the different coagulation assays.

**Coagulation assay**	**Median (IQR)**
ACT (s), *n* = 500	181 (118–301)
aPTT (s), *n* = 496	59.9 (35.6–135.6)
Anti-factor Xa (IU/ml), *n* = 455	0.26 (0.09–0.68)
CT-INTEM (s), *n* = 409	277 (164–856)
CT-Diff (s), *n* = 380	56 (−148 to 560)

*ACT, activated clotting time; aPTT, activated partial thromboplastin time; CT-INTEM, clotting time in INTEM; CT-Diff, clotting time in INTEM minus clotting time in HEPTEM; IQR, interquartile range*.

**Table 2:** Spearman correlation coefficients between heparin dose and the different coagulation tests.

	**ACT**	**aPTT**	**Anti-factor Xa**	**CT-INTEM**	**CT-Diff**
**r**_***s***_	0.19	0.20	0.38	0.03	0.17
***p*** **-value**	** <0.0001**	** <0.0001**	** <0.0001**	0.46	0.0006

*ACT, activated clotting time; aPTT, activated partial thromboplastin time; CT-INTEM, clotting time in INTEM; CT-Diff, clotting time in INTEM minus clotting time in HEPTEM; r_*s*_, Spearman's rank correlation coefficient*.

*The bold values symbolize that the value is statistically significant*.

### Thrombotic Complications

Table 3 shows the median values of the analyzed coagulation assays in the group of patients with thrombotic complications compared to the group of patients without thrombotic complications. Anti-factor Xa assay was the only test which was statistically significant between the groups and was lower in the group with thrombotic complications.

**Table 3:** Comparison of the median values from heparin dose and coagulation assays in the group of patients with and without thrombotic complications.

	**Thrombotic**	**No thrombotic**	***p*-value**
	**complications^a^**	**complications^a^**	
Heparin dose (IU/kg/h)	21.2 (0–60.6)	20.6 (0–161.3)	0.39
ACT (s)	185 (125–999)	182 (118–999)	0.85
aPTT (s)	57.6 (36.7–151)	60.8 (28.7–151)	0.89
Anti-factor Xa (IU/ml)	0.23 (0.09–0.78)	0.27 (0.09–1.9)	**0.002**
CT-INTEM (s)	267 (195–1881)	286 (154–5126)	0.14
CT-Diff (s)	49 (−148 to 1597)	60 (−126 to 4785)	0.07

*ACT, activated clotting time; aPTT, activated partial thromboplastin time; CT-INTEM, clotting time in INTEM; CT-Diff, clotting time in INTEM minus clotting time in HEPTEM*.

a *Median values and interquartile ranges*.

*The bold values symbolize that the value is statistically significant*.

### Hemorrhagic Complications

A comparison of the median values of the different coagulation tests between the group of patients who experienced a bleeding complication and the patients without bleeding complication is shown in Table 4. aPTT was the only test that was statistically significant between the two groups and was higher in the group with clinically relevant hemorrhagic complications.

**Table 4:** Comparison of the median values from heparin dose and coagulation assays in the group of patients with and without bleeding complications.

	**Bleeding**	**No bleeding**	***p*-value**
	**complications^a^**	**complications^a^**	
Heparin dose (IU/kg/h)	26.3 (0–44)	20.6 (0–161.3)	**0.03**
ACT (s)	185 (141–999)	182 (118–999)	0.90
aPTT (s)	69.4 (35.1–151)	59.8 (28.7–151)	**0.01**
Anti-factor Xa (IU/ml)	0.28 (0.09–0.75)	0.27 (0.09–1.9)	0.42
CT-INTEM (s)	297 (204–1235)	279 (154–5126)	0.15
CT-Diff (s)	61 (−46 to 960)	58 (−148 to 4785)	0.6

*ACT, activated clotting time; aPTT, activated partial thromboplastin time; CT-INTEM, clotting time in INTEM; CT-Diff, clotting time in INTEM minus clotting time in HEPTEM*.

a *Median values and interquartile ranges*.

*The bold values symbolize that the value is statistically significant*.

### Disseminated Intravascular Coagulation

The median values of the coagulation assays when DIC occurred compared to median values of the tests in the absence of DIC are represented in Table 5. No statistically significant differences in ACT values were found, whereas the median values of anti-factor Xa test were significantly lower when DIC occurred. The same could be observed with the heparin dose. In contrast, the median values of aPTT were significantly higher. No differences in the thromboelastometric tests were shown.

**Table 5:** Comparison of the median values from heparin dose and coagulation assays in the presence and absence of disseminated intravascular coagulation.

	**DIC^a^**	**Absence of DIC^a^**	***p***
Heparin dose (IU/kg/h)	15.1 (0–37.2)	20.8 (0–161)	**0.004**
ACT (s)	189.5 (143–289)	182 (118–999)	0.27
aPTT (s)	64.7 (47.4–151)	60.2 (28.7–151)	**0.04**
Anti-factor Xa (IU/ml)	0.16 (0.09–1.2)	0.27 (0.09–1.9)	** <0.0001**
CT-INTEM (s)	298 (237–1157)	278 (154–5126)	0.13
CT-Diff (s)	56 (−148 to 788)	58 (−126 to 4785)	0.60

*ACT, activated clotting time; aPTT, activated partial thromboplastin time; CT-INTEM, clotting time in INTEM; CT-Diff, clotting time in INTEM minus clotting time in HEPTEM; DIC, disseminated intravascular coagulation*.

a *Median values and interquartile ranges*.

*The bold values symbolize that the value is statistically significant*.

## Discussion

In the present study, we evaluated the correlation of an administrated heparin dose to different coagulation assays in newborns with CDH undergoing venoarterial ECMO and assessed whether the different coagulation assays correlated with thrombotic and/or hemorrhagic complications, when the management of anticoagulation was based only on ACT values. Anti-factor Xa assay showed a moderate correlation with heparin dose, whereas the other tests correlated only weakly. The levels of anti-factor Xa were lower in the group with thrombotic complications, while aPTT was higher in the group with hemorrhagic complications. In patients experiencing DIC, heparin dose and anti-factor Xa levels were lower, while no differences in ACT and CT-INTEM and CT-Diff were seen.

The ACT, which is our standard assay to manage heparin administration on ECMO, showed only a weak correlation to heparin dose. This finding is consistent with prior studies of pediatric patients on ECMO (4, 11, 14–16) and may suggest that management of anticoagulation during ECMO based only on ACT measurements leads to sub-optimal anticoagulation in these patients. Although pediatric patients and neonates have physiologically prolonged aPTT values, several publications have shown a better correlation of heparin dose to aPTT compared to ACT, suggesting that the aPTT could be a more accurate test than the ACT to measure heparin effect in pediatric patients including neonates during ECMO (4, 14, 17, 18). Our findings do not support this hypothesis: the correlation of heparin doses to aPTT was only weak and not stronger than the correlation to ACT. Several studies have shown a more significant correlation of heparin dose to anti-factor Xa assay values in pediatric patients and neonates during ECMO, compared to ACT and aPTT values (4, 11, 15, 19). In our study, the anti-factor Xa assay showed the strongest correlation to heparin dose. Nankervis et al. (11) found a strong correlation of anti-factor Xa assay to the administered heparin dose (*r* = 0.75), whereas the correlation in our study was only moderate (*r* = 0.38) and comparable to the findings of Bembea et al. (15) (*r* = 0.33). In the study of Nankervis et al. (11), the median heparin dose was 42.2 IU/kg/h, and the median value of anti-factor Xa assay was 0.7 IU/ml. These values are considerably higher in comparison with the median values reported by Bembea et al. (15) (34 IU/kg/h and 0.4 IU/ml) and our values (21.2 IU/kg/h and 0.26 IU/ml). This could suggest that the correlation of anti-factor Xa assay to heparin is stronger when higher heparin doses are administered.

Although the use of viscoelastic tests to monitor coagulation on ECMO is not widespread, the literature reports benefits of their use (20). Major obstacles for the use of thromboelastometry for coagulation management in neonatal ECMO are the scarcity of data on reference ranges for ROTEM^®^ parameters in neonates and the lack of established therapeutic ranges for monitoring anticoagulation (19, 21). Panigada et al. concluded in a study of an adult population that the use of a thromboelastography-driven protocol to manage heparin anticoagulation during venovenous ECMO seemed to be feasible, not associated with an increased rate of complications, and even allowed the administration of lower heparin doses compared to the use of an aPTT-driven protocol (22). Northrop et al. (10) showed that the use of a protocol which included anti-factor Xa assays, AT measurements, and thromboelastometry was associated with a reduction of thrombotic and hemorrhagic complications in pediatric patients on ECMO. Henderson et al. (19) showed a feasible management of heparin administration using the thromboelastography in a study of pediatric patients and neonates on ECMO and established the optimal target for one thromboelastographic parameter for predicting a significant thrombotic event. In contrast to these findings, we did not find a strong correlation of the evaluated thromboelastometric parameters to heparin dose. CT-Diff correlated only weakly, and no significant correlation of CT-INTEM to heparin dose was found. The graphical representation of the obtained thromboelastometric values showed a lack of sensitivity of the tests in response to a change in the heparin dose.

Showing the strongest correlation to heparin dose is not enough to demonstrate a superiority of one coagulation assay to manage the anticoagulation during ECMO. The goal of anticoagulation during ECMO is to minimize thrombotic complications in patients and the ECMO circuit without increasing hemorrhagic complications. Therefore, in a study that compares different coagulation assays, these clinical complications should be considered.

Irby et al. (23) published the first study in 2014, which linked anti-factor Xa concentrations with clinically relevant thrombotic complications in pediatric patients during ECMO. This study suggested that higher anti-factor Xa activity levels were associated with a lower probability of requiring a change of the ECMO circuit. In our study, only anti-factor Xa assay out of the evaluated tests showed statistically significant values between groups with and without thrombotic complications. Because heparin was still being administered according to ACT values, median values of anti-factor Xa assay were below 0.3 IU/ml in both groups. According to the current literature, such values are in a sub-therapeutic range (3). Nevertheless, the median value of anti-factor Xa assay was significantly lower in the group with clinically relevant thrombotic complications in comparison with the group without thrombotic complications. This observation would potentially suggest that dosing heparin according to anti-factor Xa assay values would not only lead to higher anti-factor Xa assay values but also to a greater difference in terms of thrombotic complications.

When considering hemorrhagic complications, only aPTT out of all evaluated coagulation assays was significantly higher in the group with clinically relevant bleeding in comparison with the group without hemorrhagic complications. The median dose of heparin infusion was also significantly higher in the group with hemorrhagic complications. This finding could suggest that monitoring aPTT could be important to prevent bleeding complications. In a systematic review, Willems et al. (24) compared time-guided vs. anti-factor Xa-guided anticoagulation strategies for UNFH titration in patients on ECMO. This meta-analysis showed that an anti-factor Xa-based anticoagulation strategy was associated with fewer hemorrhagic complications without an increase in thrombotic events, when compared to a time-based anticoagulation strategy (24). In contrast to these findings, we found no differences regarding anti-factor Xa values, when comparing the groups with and without bleeding events. However, we did not compare two different anticoagulation strategies. Nevertheless, the results shown in the study by Willems et al. (24) could suggest that an anti-factor Xa-based UNFH titration strategy could also reduce bleeding complications in neonatal patients on ECMO.

Values of ACT and aPTT are typically elevated in the setting of DIC. If heparin is titrated according to ACT values, it will need to be reduced to keep ACT in the target values. This finding is demonstrated in our study: while median values of ACT showed no significant differences, the median of the heparin dose and the median value of anti-factor Xa assay were significantly lower in the setting of DIC. In contrast, the median value of aPTT was significantly higher. No difference was observed by the thromboelastometric parameters CT-INTEM and CT-Diff. This finding supports that anti-factor Xa assay correlates better to heparin dose than ACT, aPTT, and the two evaluated parameters of the thrombelastometry. The clinical correlations of ACT, aPTT, and anti-factor Xa in the setting of DIC are relatively unknown and should be investigated in future studies (4).

Our homogenous study population, comprising only CDH patients, offered several advantages. First, confounders derived from the differences in hemostatic system between neonates and older children were avoided. Furthermore, general inflammation, as present in sepsis or pneumonia, which might affect coagulation, interfering and influencing the anticoagulation management during ECMO, was excluded.

There are several limitations to our study. First, even though we have a number of measurements conducted, the sample size is still small. Also, results may not be applicable to older children supported by ECMO, because of developmental hemostasis in neonates. A second issue is that the design of the study does not allow declaration of superiority and/or inferiority of one of the coagulation assays. In our study, anticoagulation was managed based on ACT values, and the other assays were only measured but not used for the management of anticoagulation. However, our results are consistent with prior studies in pediatric patients on ECMO. A prospective study to evaluate if managing anticoagulation based on anti-factor Xa assay measurements instead of ACT could reduce thrombotic and bleeding complications in neonates during ECMO is currently being conducted at our institution.

## Conclusions

Anti-factor Xa correlates stronger to heparin dose than ACT and could be a more accurate method to dose anticoagulation with heparin in neonates during ECMO. Moreover, managing ECMO anticoagulation based on anti-factor Xa assay measurements instead of ACT could reduce thrombotic and bleeding complications in these patients. Nevertheless, the moderate correlation of anti-factor Xa assay to heparin dose indicated that it is not the ideal test. Furthermore, the anti-factor Xa assay does not provide any information about the status of the patient's hemostasis. To allow an optimal management of the anticoagulation during ECMO and to avoid complications, additional tests which can assess the status of the patient's hemostasis are probably necessary. The use of thromboelastometry does not seem to have any additional benefit for dosing heparin in neonates supported with ECMO. However, due to the potential information that viscoelastic tests can provide, the use of thromboelastometry in pediatric and neonatal ECMO should be further explored.

## Data Availability Statement

The raw data supporting the conclusions of this article will be made available by the authors, without undue reservation.

## Ethics Statement

The studies involving human participants were reviewed and approved by Ethics committee of the Medical Faculty Mannheim of the University of Heidelberg (Ethics Committee II). Written informed consent to participate in this study was provided by the participants' legal guardian/next of kin.

## Author Contributions

AP, CD, CW, SH, TS, and NR contributed to the concept and design, acquisition, interpretation of data, and drafting of the article. TJ and TD contributed to the interpretation of data and revised the article for important intellectual content. All authors approved the final version of the article.

## Funding

This study was supported by a research grant from the German ECMO booster club (Förderverein ECMO Deutschland e.V.).

## Conflict of Interest

The authors declare that the research was conducted in the absence of any commercial or financial relationships that could be construed as a potential conflict of interest.

## Publisher's Note

All claims expressed in this article are solely those of the authors and do not necessarily represent those of their affiliated organizations, or those of the publisher, the editors and the reviewers. Any product that may be evaluated in this article, or claim that may be made by its manufacturer, is not guaranteed or endorsed by the publisher.
